# Role of a radiopaque agent and surveillance radiographs for peripherally inserted central catheters in newborn infants

**DOI:** 10.1007/s00247-023-05705-7

**Published:** 2023-07-25

**Authors:** Yulia Stekhova, Vinayak Kodur, Gemma Lowe, Jane Baird, Krista Lowe, James Elhindi, Rajesh Maheshwari, Dharmesh Shah, Daphne D’Cruz, Melissa Luig, Pranav R. Jani

**Affiliations:** 1 Department of Neonatology, Westmead Hospital, Darcy Road, Westmead, NSW 2145 Australia; 2 Research and Education Network, Westmead Hospital, Westmead, NSW Australia; 3 The Reproduction and Perinatal Centre, Faculty of Medicine and Health, The University of Sydney, Sydney, NSW Australia; 4 The Faculty of Medicine and Health, The University of Sydney, Sydney, NSW Australia

**Keywords:** Infant, Neonatal intensive care unit, Peripherally inserted central catheter, Radiograph, Radiopaque agent

## Abstract

**Background:**

Controversy exists regarding the use of a radiopaque agent to identify peripherally inserted central catheter (PICC) tip positions in newborn infants and of serial radiography to monitor PICC tip migration.

**Objective:**

To investigate the roles of (1) the injection of a radiopaque agent to identify PICC tip position and (2) the performance of weekly radiography to monitor PICC migration.

**Materials and methods:**

This retrospective single-centre cohort study included newborn infants who received a PICC between 1 January 2016 and 31 December 2020. A radiopaque agent was injected to identify PICC tip position and radiographs were performed weekly to detect PICC migration.

**Results:**

We identified 676 PICC episodes in 601 infants. A radiopaque agent was used for 590 of these episodes. There was no difference in the proportion of central PICC tip positions based on radiopaque agent use status (490/590, 83% for the radiopaque agent used group versus 73/85, 85.8% for the radiopaque agent not used group, *P*=0.51). Irrespective of the site of PICC insertion, outward migration was observed for most centrally placed PICCs over their entire in situ duration. Inward migration was identified in 23 out of 643 PICC episodes (3.6%) only on radiographs obtained on or before day 7. Based on serial radiographs, the odds for PICC tips remaining in a central position were lower the longer the PICC remained in situ (adjusted odds ratio-OR 0.93; 95% confidence interval 0.92–0.95). There was no difference in PICC migration between side and limb of insertion.

**Conclusion:**

PICC tips can be identified without injection of a radiopaque agent. Serial radiographs identified PICC migration over the in situ duration. This study has implications for reducing exposure to a radiopaque agent and ongoing migration surveillance practices.

**Graphical abstract:**

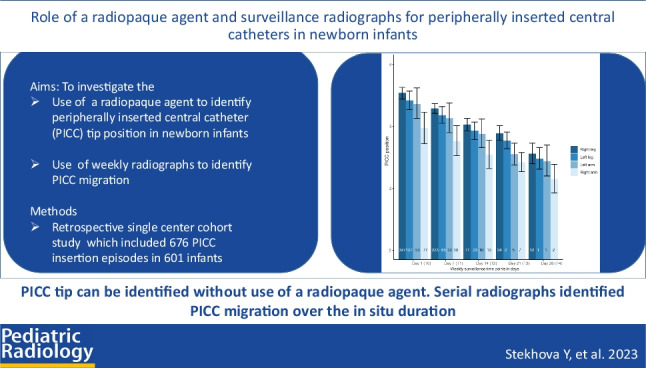

## Introduction

Peripherally inserted central catheters (PICCs) are commonly used in neonatal intensive care units (NICUs). They allow the timely administration of parenteral nutrition and medications to unwell newborn infants [1, 2]. After insertion of a PICC and for the in situ duration, it is essential that the catheter tip should lie in a large central vein. For PICCs inserted into the upper limb, this position is at the junction of the superior vena cava and the right atrium and for PICCs inserted in the lower limb, this position is in the thoracic inferior vena cava between T8 and T12 [1, 3–5]. PICCs that are not positioned in a central vein may be at an increased risk of developing complications such as pericardial tamponade, neurological complications from accidental cannulation of the ascending lumbar vein, catheter-related bloodstream infection and mechanical failure [4–6]. The inward or outward migration of PICC tips can occur either soon after their insertion or at any time while in situ [7–10]. Inward catheter migration can cause complications such as cardiac tamponade or arrhythmias and outward catheter migration (catheter tip position in a non-central vein) can cause complications such as thrombosis, extravasation and vascular injury [11–13]. Therefore, timely detection of PICC malposition and migration is imperative to prevent these serious complications.

Controversy exists regarding both the injection of a radiopaque agent through the PICC immediately after insertion to locate the tip and the utility of serial radiography (i.e. no radiopaque agent) over the in situ duration to detect PICC migration. Some researchers have suggested routine use of a radiopaque agent for accurately locating PICC tip position and performing serial radiographs to identify PICC migration [4, 9, 10, 14–16]. In our unit, we follow both these practices.

The aims of this study were to investigate the use of (1) injection of a radiopaque agent for locating PICC tip position and (2) weekly serial radiography (without using a radiopaque agent) to identify PICC migration. We hypothesized that the routine use of both these practices may not be necessary. 

## Materials and methods

**Fig. 1:**
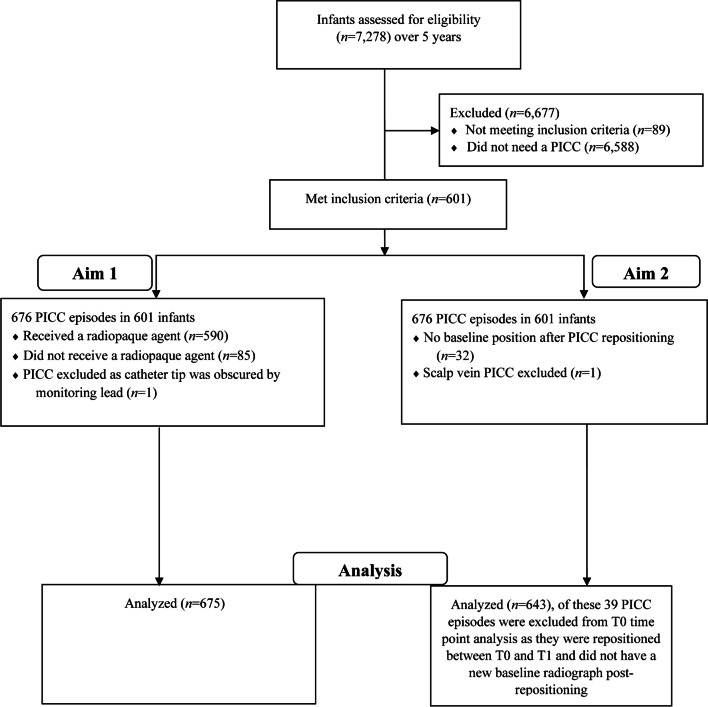
Flow diagram. *PICC* peripherally inserted central catheter, *T0* day of insertion, *T1* 1 week following insertion

Ethical approval was obtained from the Western Sydney Local Health District, Sydney, Australia, for this single-centre retrospective cohort study. All infants born between 1 January 2016 and 31 December 2020 and admitted to the neonatal intensive care unit (NICU) at Westmead Hospital in Sydney, Australia, were eligible to participate. Infants who had a PICC were identified from a prospectively maintained and verified Neonatal Intensive Care Units’ (NICUS) database. This database collates the maternal, perinatal and neonatal clinical data of newborn infants admitted to all tertiary NICUs in New South Wales and the Australian Capital Territory. The data are collected and verified by designated audit officers following standardized definitions for clinical outcomes. Infants with a PICC in situ who were transferred to other hospitals or who had a PICC inserted at other hospitals and were then transferred to our NICU were excluded, due to lack of data completeness (Fig. 1). Demographic, PICC procedure-related and radiological data of eligible infants were obtained from the NICUS database and electronic medical records.

### Peripherally inserted central catheter insertion and maintenance practice

PICC insertion and maintenance was practised as per the unit’s guideline. PICC use was reserved for infants requiring total parenteral nutrition or prolonged (>7 days) parenteral antibiotic therapy. In our unit, three types of polyurethane PICCs were available for use: single lumen Premicath® 1Fr (28G), single lumen Nutriline® 2Fr (24G) and double lumen Twinflo® 2Fr (24G) (all three from Vygon Gesellschaft mit beschränkter Haftung & Compagnie Kommanditgesellschaft, Aachen, Germany). For infants  <1,000 g at birth, a 1-Fr PICC was used. All PICCs were inserted by an operator skilled in the technique (either a neonatal nurse practitioner, senior registrar, neonatal fellow or consultant). Where possible, two team members were present for the PICC insertion procedure; one was the primary proceduralist and the second assisted with the procedure. PICCs were inserted through either the basilic or cephalic vein into the superior vena cava or through the long saphenous vein into the inferior vena cava. External anatomical landmarks were used to determine the length of the PICC insertion. For the upper limb, this length was the distance in centimetres between the insertion site and the jugular notch of the sternum. For the lower limb, the length in centimetres was the distance between the insertion site and the xiphisternum [17]. Excess catheter beyond the insertion site was looped over the skin, ensuring that the catheter was not kinked. The PICC was secured to the skin using Steri-Strip™ adhesive tapes (3 M Company, St Paul, MN). A clear semi-permeable dressing was placed over the insertion site.

**Fig. 2:**
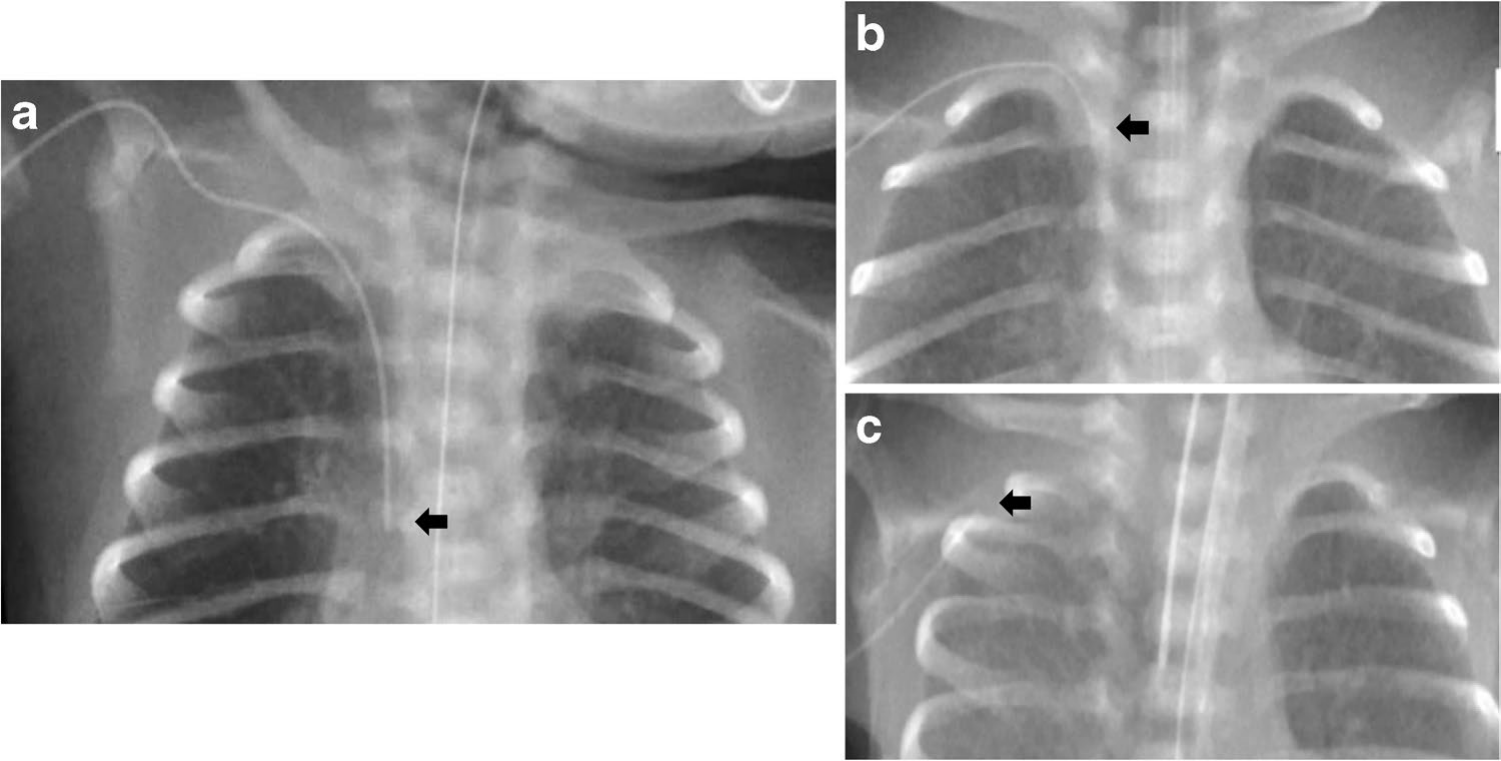
Position of peripherally inserted central catheter (PICC) tips in the upper limb. **a** A 5-day-old girl. Anteroposterior (AP) chest radiograph shows a PICC tip in a central ideal position—catheter tip between the 3rd and the 5th thoracic vertebrae, which corresponds to the superior vena cava and the cava-atrial junction (*arrow*). **b** A 1-week-old girl. AP chest radiograph shows a PICC tip in a central accept position (*arrow*): catheter tip between the medial third of the clavicle and 2nd thoracic vertebra (subclavian or brachiocephalic vein). **c** A 3-week-old boy. AP chest radiograph shows a PICC tip beyond the medial third of the clavicle (*arrow*)

**Fig. 3:**
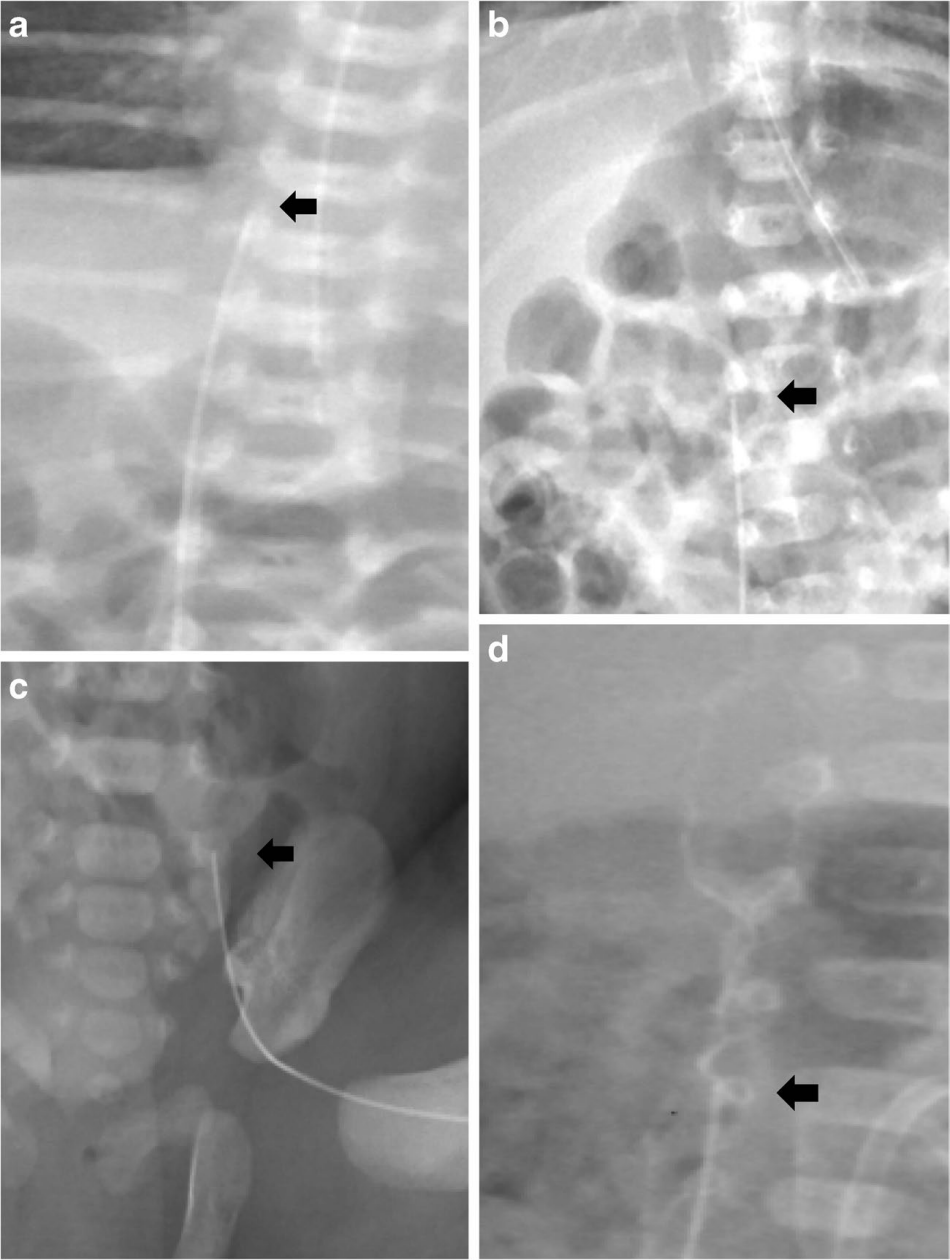
Position of peripherally inserted central catheter (PICC) tips in the lower limb. **a** A 3-day-old boy. Anteroposterior (AP) chest and abdomen radiograph shows  a PICC tip in a central ideal position—catheter tip between the 8th and the 12th thoracic vertebrae (*arrow*), which corresponds to the inferior vena cava. **b** A 4-week-old girl. AP chest and abdomen radiograph shows a PICC tip in a central accept position: catheter tip between the 1st and the 3rd lumbar vertebrae (*arrow*). **c** A 6-day-old girl. AP chest and abdomen radiograph shows a PICC tip beyond the 3rd lumbar vertebra (*arrow*). **d** A 3-day-old boy. AP chest and abdomen radiograph shows a PICC tip in a non-central position with radiopaque agent in the ascending lumbar plexus (*arrow*)

For most patients, the PICC tip position was then confirmed by injecting a small amount (0.2 mL to 0.3 mL) of a radiopaque agent (Omnipaque 240, Iohexol equivalent to iodine 240 mg/mL, GE Healthcare, Chicago, IL) using a 10 mL syringe (as per the manufacturer’s instructions), while simultaneously taking the radiograph. After taking the radiograph, the radiopaque agent was flushed into the patient. The dead space for these PICC lines ranges from 0.09 mL (in 20 cm Premicath®) to 0.2 mL (in 30 cm Twinflo®). The decision to use a radiopaque agent for PICC tip location was not based on random allocation but at the discretion of different providers. For this study (as shown in Figs. 2 and 3), a central ideal position of the PICC tip (in a central vein, but outside the cardiac silhouette) was defined as follows: (i) for PICCs inserted in the arm: PICC tip between the 3rd and the 5th thoracic vertebrae, which corresponds to the superior vena cava and the cava-atrial junction and (ii) for PICCs inserted in the leg: PICC tip between the 8th and the 12th thoracic vertebrae, which corresponds to the inferior vena cava. Where the PICC tip was between the medial third of the clavicle and 2nd thoracic vertebra (subclavian or brachiocephalic vein) or between the 1st and the 3rd lumbar vertebrae, this was referred to as a central acceptable position. Both central ideal and central acceptable positions were considered acceptable regardless of the infusate to be used (usually we administer total parenteral nutrition or antibiotics through the PICCs). All positions of the PICC tip beyond the central ideal or central acceptable were considered as non-central in position. This also included location of the PICC tip in a peripheral vein. After PICC insertion, in order to ascertain PICC migration, weekly surveillance radiographs were performed for all infants, without using a radiopaque agent. Radiographs on the day of insertion were labelled as T0, a week after insertion as T1 and so on. Radiographic images obtained at the time of PICC insertion and for weekly surveillance for all patients were reported by the institution’s radiologist on service. The radiographs were acquired using MobileDaRt X-ray System (Shimadzu Corporation, Kyoto, Japan) and the images were accessed using Sectra UniView® web viewer (Sectra AB, Linkoping, Sweden). In Sectra UniView, the quality of images was optimized by using zoom in, invert and contrast functions.

### Statistics

Data analysis was performed using statistical analysis software (Stata SE Version 14.2, StataCorp, College Station, TX). All hypotheses were tested at a significance level of 0.05 with two-sided alternative hypotheses. The study cohort’s demographic and clinical characteristics are summarized by descriptive statistics. Continuous variables where normally distributed are summarized as mean and standard deviation and where not normally distributed are summarized as median and interquartile range (IQR). Categorical variables are summarized as proportion and count. Student’s *t*-test, a rank-sum test, chi-square test or Fisher’s exact test were used to assess differences in sample mean, median and proportion, respectively.

The outcomes were assessed with univariate and multivariate generalized estimating equations. The models for first outcome were adjusted for potentially confounding variables such as gestational age, birth weight, limb of insertion and side of insertion. The models for second outcome were adjusted for fixed effects of day of radiograph, side, limb, year of birth and interaction term between side and limb. Odds ratios (OR), 95% confidence intervals (CI) and *P*-values are reported. The models in the outcomes were additionally adjusted for an interaction parameter between side and limb. The goodness-of-fit of the interaction parameter was assessed by a Wald test, with *P*-values calculated by a Monte Carlo simulation, and measured by the quasi-likelihood information criterion. Throughout, generalized estimating equations handled missing data automatically by adjusting appropriate variance and covariance estimates. A pragmatic study duration of 5 years was chosen for sample size calculation based on the NICU’s admission rate.

## Results

### Patient and peripherally inserted central catheter characteristics

We identified 601 infants who were born between 1 January 2016 and 31 December 2020. The median gestational age of the cohort was 29 weeks (IQR 27–31) and median birthweight was 1,200 g (IQR 960–1,455). Male infants represented 54% (326/601). Over the duration of the study, we screened 7,278 infants who were admitted to the NICU for their eligibility. Of these, 6,677 infants were excluded: 6,588 infants did not need a PICC and 89 infants either had a PICC insertion at another hospital and were transferred to our unit or PICC was inserted in our unit, but these infants were transferred to another hospital before PICC removal. For the final analysis, we included 676 PICC insertion episodes in 601 infants. The most common indication for PICC insertion (546/676, 80.8%) was for the administration of parenteral nutrition in view of prematurity. A single lumen PICC (550/676, 81.4%) and a 1-Fr catheter (481/676, 71.2%) were most commonly used. The median PICC in situ time was 9 days (IQR 6–14). Of the 676 PICC episodes, 143 (21.2%) were placed in the arm, 532 (78.7%) in the leg and one in a scalp vein. Of the 143 PICCs inserted in the upper limb, 86 (60.1%) were inserted in the right arm (basilic or cephalic vein in the cubital fossa) and 57 (39.9%) in the left arm. Of the 532 PICCs inserted in the leg, 401 (75.4%) were placed in the right leg and 131 (24.6%) in the left leg. For the PICC tip positions, 563/675 (83%) were central. Of the central PICC tip positions, 103/563 (18.3%) were inserted in the arm and 460/563 (81.7%) were inserted in the leg.

### Relationship between radiopaque agent use and the peripherally inserted central catheter tip position

**Fig. 4:**
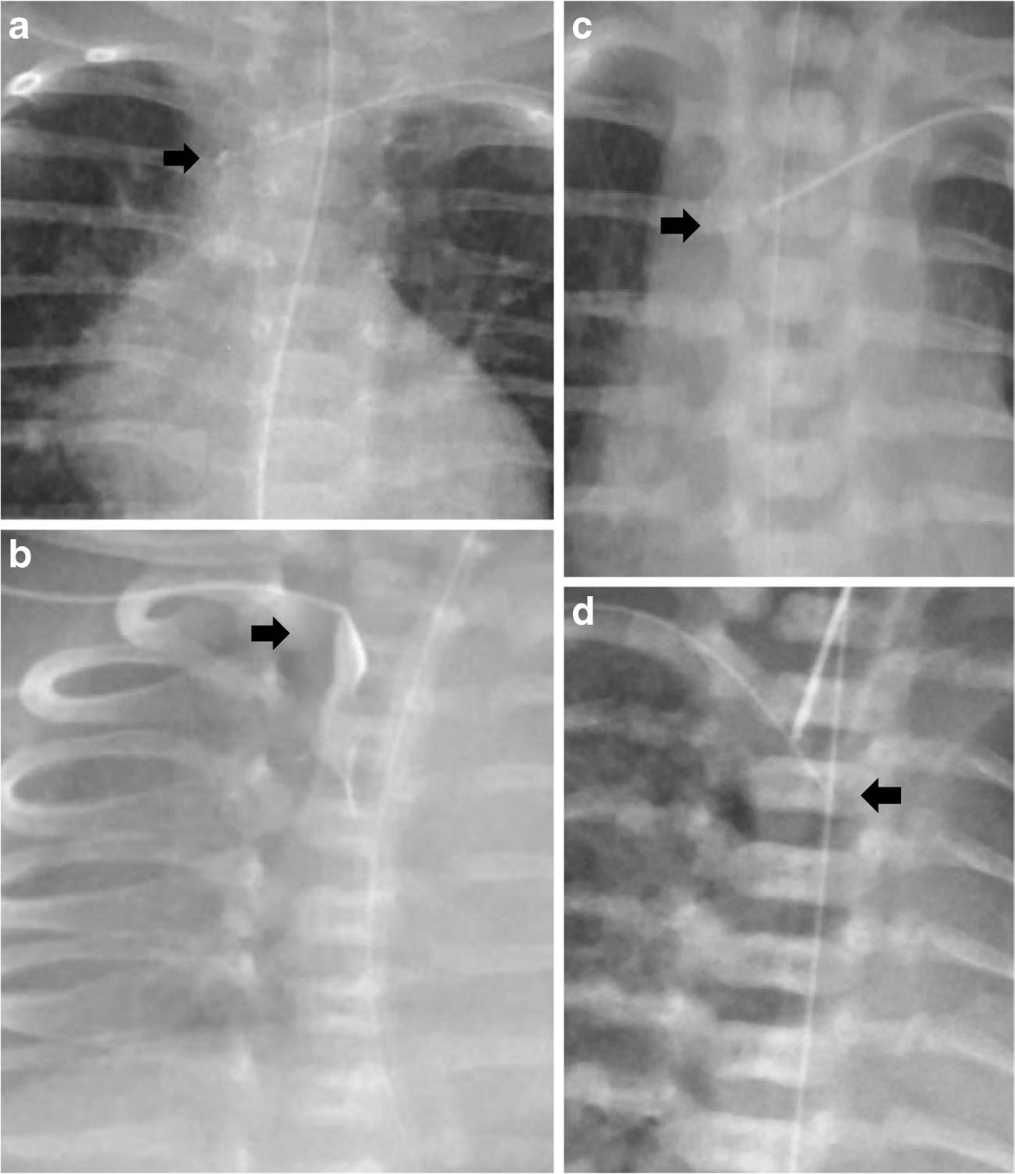
Visualization of 1 and 2 French (Fr) catheter tips with (**a** and **b**) and without (**c** and **d**) using a radiopaque agent. **a** A 4-day-old boy. Anteroposterior (AP) chest radiograph shows a 1 Fr peripherally inserted central catheter (PICC) with radiopaque agent (*arrow*). **b** A 10-day-old girl. AP chest radiograph shows a 2 Fr PICC with radiopaque agent (*arrow*). **c** A 4-week-old boy. AP chest radiograph shows a 1 Fr PICC without radiopaque agent (*arrow*). **d** A 5-week-old boy. AP chest radiograph shows a 2 Fr PICC without radiopaque agent (*arrow*)

**Fig. 5:**
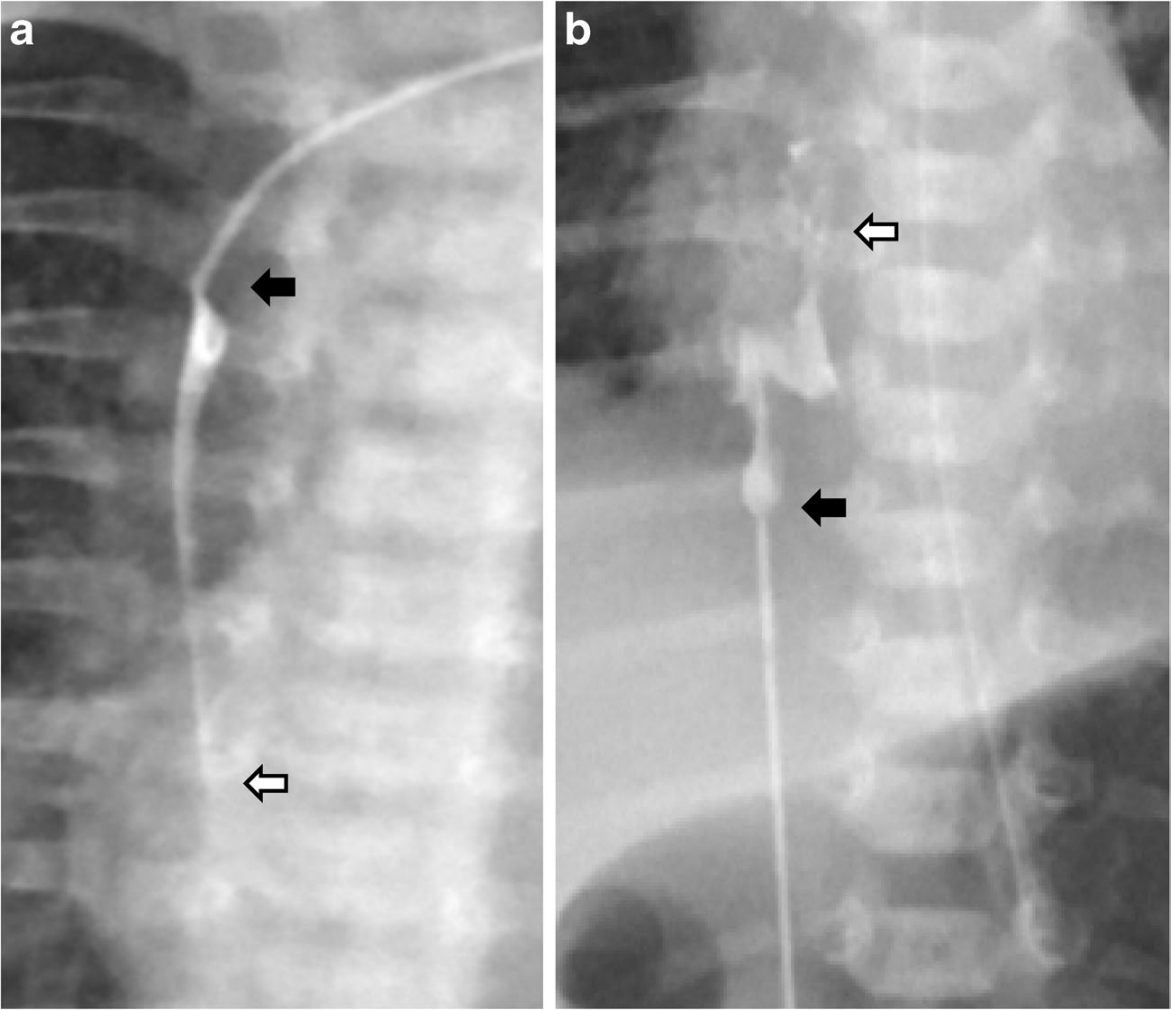
Radiographs show radiopaque agent jets (*white arrows*) beyond the peripherally inserted central catheter (PICC) tips (*black arrows*). **a** A 3-day-old girl. AP chest radiograph of upper limb PICC. **b **a 5-day-old boy. AP chest and abdomen radiograph of lower limb PICC

The cohort was dichotomized based on the use of a radiopaque agent. Of the 676 PICC insertion episodes, one episode was excluded because the PICC tip position could not be confirmed as it was obscured by a monitoring lead. Of the 675 PICC insertion episodes, a radiopaque agent was used for 590 (87%). Most baseline clinical characteristics were similar between the two groups (Table 1) except for birthweight and gestational age. There was no difference between the proportion of central PICC tip position (central ideal and central acceptable) based on radiopaque agent use status (radiopaque agent used group: 83.1% compared to no radiopaque agent used group: 85.9%, *P*=0.51). For non-central lower limb PICC positions, using a radiopaque agent identified PICC tip in the ascending lumbar vein (6/72, 8.3%) (Fig. 3). For all infants where a radiopaque agent was not used, the PICC tip position could be easily identified on the radiographs irrespective of central or non-central position and PICC caliber (Fig. 4). Significantly more radiographs were required in the radiopaque agent group to confirm the PICC tip position compared to no radiopaque agent used group (Table 1). In these cases, the radiopaque agent jet was seen beyond the PICC tip (Fig. 5) and these PICCs required repositioning to optimize their location.

**Table 1:** Differences in the cohort characteristics based on radiopaque agent use

Clinical characteristic	Radiopaque agent used (*n*=590)	Radiopaque agent not used (*n*=85)	*P*-value
Birthweight, grams (median and IQR)	1,152.5 (900–1,440)	1,245 (1,050–1,410)	0.04
Gestational age, weeks (median and IQR)	29 (28–32)	29 (27–31)	0.01
PICC insertion in the first week of life	517 (87.6%)	80 (94.1%)	0.12
Appropriate for gestational age	445 (75.4%)	65 (76.5%)	0.48
Male sex	323 (54.7%)	45 (52.9%)	0.76
Side of PICC insertion			
Left	163 (27.6%)	25 (29.4%)	0.73
Right	427 (72.4%)	60 (70.6%)	
Limb of PICC insertion			
Arm	131 (22.3%)	11 (12.9%)	0.05
Leg	457 (77.7%)	74 (87.1%)	
PICC tip position			
Central	490 (83.1%)	73 (85.9%)	0.51
Lower limb	394 (80.4%)	66 (90.4%)	
Upper limb	96 (19.6%)	7 (9.6%)	
Not central	100 (16.9%)	12 (14.1%)	
Number of radiographs performed for PICC tip confirmation			
1	328 (55.6%)	65 (76.5%)	<0.001
2	198 (33.6%)	19 (22.3%)^a^	
≥ 3	64 (10.8%)	1 (1.2%)^a^	
PICC calibre^b^			
1Fr (28 Gauge)	442 (74.9%)	67 (78.8%)	0.95
2 Fr (24 Gauge)	134 (22.7%)	18 (21.2%)	

*Fr* French,; *IQR* inter quartile range, *PICC* peripherally inserted central catheter

^a^ >1 radiograph performed to optimize PICC positioning rather than for localizing PICC tip

^b^Not documented in 14 patients

Binary univariate and multivariable logistic regression models were performed for the clinical variables associated with PICC tip position. The birth year and limb used for PICC insertion when adjusted for gestational age and birthweight were the two significant variables. The OR for a central position of the PICC tip was higher for PICCs inserted in 2020 compared to 2016 (adjusted [a]OR 1.49, 95% CI 1.26–1.76). The OR for a central position (central ideal and central acceptable) of the PICC tip was lower for left arm (aOR 0.39, 95% CI 0.19–0.76), right arm (aOR 0.37, 95% CI 0.21–0.66) and left leg (aOR 0.55, 95% CI 0.32–0.95) compared to right leg (arbitrarily chosen as the reference) after adjusting for gestational age, birthweight and infant’s birth year.

### Weekly surveillance for peripherally inserted central catheter migration using radiographs

**Fig. 6:**
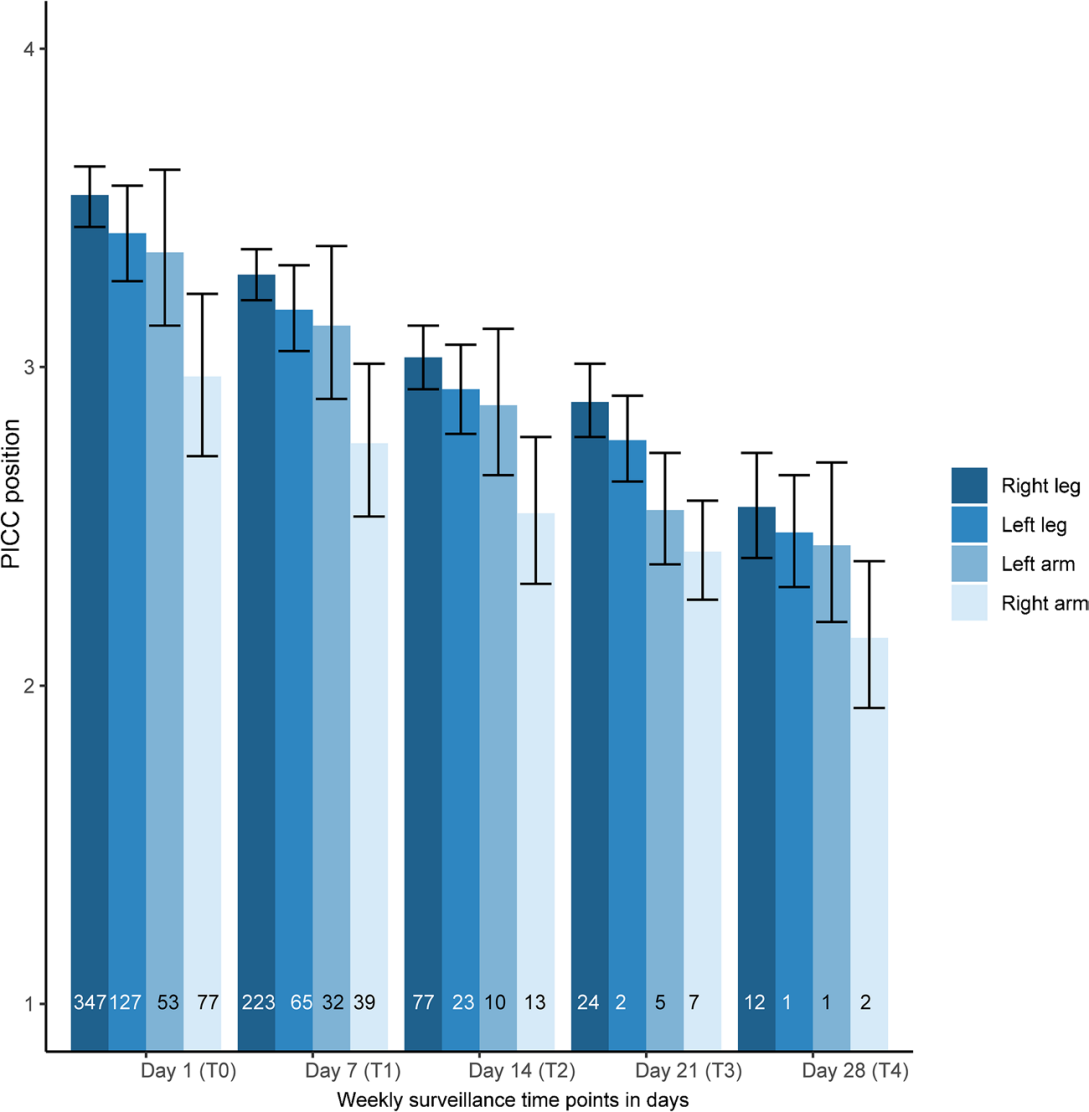
Side- and limb-based trends in peripherally inserted central catheter (PICC) migration over time from a central to a peripheral position. Number within the bar indicates the count of PICC episodes based on the limb of insertion. The odds for PICC tips remaining in a central position were lower the longer the PICC remained in situ (adjusted odds ratio 0.93; 95% confidence interval 0.92–0.95). We excluded 39 PICC episodes from time 0 (T0) analysis as they were repositioned between time 0 (T0) and time 1 (T1) and they did not have a new baseline radiograph post-repositioning. *4* central ideal position, *3* central acceptable position, *2* non-central position, *1* peripheral position, *T0* day of insertion, *T1* 1 week following insertion, *T2* 2 weeks following insertion, *T3* 3 weeks following insertion, *T4* 4 weeks following insertion

For the second aim of our study, from the 676 PICC insertion episodes, one was excluded as it was inserted in a scalp vein. Additionally, 32 PICC insertion episodes were excluded as a repeat radiograph was not performed after PICC repositioning, therefore, their true baseline position at T0 could not be verified. A total of 643 PICC episodes were included for the final analysis. There were 359, 123, 38 and 16 PICC lines screened on day 7 (T1), day 14 (T2), day 21 (T3) and day 28 (T4), respectively. The trend in PICC migration over time based on side and limb of PICC insertion is shown in Fig. 6. Most PICCs, irrespective of their insertion sites, migrated over the in situ duration. Inward migration was identified in 23 out of 643 PICC episodes (3.6%) at day 7 (T1). Of these, 18 PICCs were inserted in the arm and only five of them required repositioning. It was also noted that in 11 out of 18 (61.1%) PICCs the arms were lifted above the head on the follow-up radiographs, therefore likely contributing to an inward PICC line migration. No PICCs migrated inwards beyond day 7. As shown in Fig. 6, PICCs migrated outward over the in situ duration. If the PICC tip was identified to be in a non-central position on follow-up radiographs, its use was at the discretion of the attending physician. Where the PICC was left in situ, it was closely monitored for the occurrence of complications. PICC tip could be identified on all weekly surveillance radiographs, whether a radiopaque agent was used or not at the time of PICC insertion. The adjusted OR for PICC tip remaining in a central position were lower the longer the PICC remained in situ (adjusted OR 0.93; 95% CI 0.92–0.95). There was no difference in PICC migration between left- and right-sided PICCs (independent of the limb of insertion). PICCs in the leg had greater odds of being centrally located compared to PICCs in the arms (aOR 1.20, 95% CI 1.13–1.26). We examined the interaction between the side and limb of PICC insertion and found that PICCs inserted in the left leg had a lower probability of being centrally located compared to PICCs inserted in the right leg (aOR 0.91, 95% CI 0.83–0.99). PICCs inserted in 2020 had a higher probability of remaining in a central position compared to PICCs inserted in 2016 (aOR 1.21, 95% CI 1.16–1.26). The year of birth (insertion) impacted the position of the PICC tip independent of in situ time.

## Discussion

We aimed to investigate the injection of a radiopaque agent to identify PICC tip position and the clinical utility of performing weekly serial radiographs for identifying PICC migration. We observed that PICC tip can be identified without injecting a radiopaque agent when it is in a central position. However, injecting a radiopaque agent identified the specific location of malposition (in the ascending lumbar plexus) for the non-central lower limb PICCs. Weekly surveillance using radiographs identified that PICCs migrated over the in situ duration. Only a small proportion of PICCs migrated inwards on day 7 radiographs. PICCs inserted in the right leg were most likely to remain in a central position.

Contrary to our results, Reece et al. reported that in half of their study cohort the PICC tip position could not be accurately determined on a radiograph and for those PICCs, the tip was identified after using a radiopaque agent [14]. Therefore, they concluded the need for routine use of a radiopaque agent for identifying PICC tip position. Although they used silicon- and polyurethane-based PICCs, the authors did not report on the visibility of PICC based on the type of PICC material. Odd et al. reported on improved likelihood of visualizing a PICC tip by using a radiopaque agent, although all PICC tips could not be identified [18]. In our study, we could visualize PICC tips on all radiographs. Possible reasons for this observation include technical improvement in the manufacture of the PICCs that are inserted in newborns and improved visibility of PICCs using modern radiography equipment. Therefore, institutions with skills in PICC insertion should consider not injecting a radiopaque agent routinely for identifying PICC tip position. Interestingly, PICCs in the radiopaque agent used group were repositioned more often compared to PICCs in the no radiopaque agent used group; therefore, infants required more radiographs to confirm the final PICC tip position. This was attributed to the radiopaque agent ejecting beyond the PICC tip and filling the blood vessel. This may have caused difficulty in differentiating the tip of the radiopaque agent jet from the PICC tip. For those cases, we did not collect details regarding PICC insertion-related technical complexities.

Perhaps radiopaque agent use could be reserved for PICCs that are either in a non-central location or when the tip location cannot be identified on radiographs. This might reduce the exposure of unwell newborn infants to a radiopaque agent, reduce the possible side-effects from radiopaque agent exposure (which remain unknown), potential staff time savings and reduce the cost to the organization from its routine use. On the other hand, it is possible that some newborn infants may need additional radiographs using a radiopaque agent to accurately locate the PICC tip position when the PICC tip cannot be seen on a radiograph. Additionally, recent literature shows that ultrasound can offer timely and accurate identification of the PICC tip, thus potentially reducing the number of radiographs and limiting radiation exposure [19–21].

PICC migration has been reported to occur in the first 24–72 h after insertion [4, 9, 10, 22]. Therefore, some researchers have suggested performing a repeat radiograph 24 h after inserting a PICC to identify migration, while others have suggested performing imaging surveillance up to 72 h after PICC insertion [4, 8]. Although the practice of performing surveillance radiographs has been reported by some, the quality of evidence to support this practice is lacking [22]. To the best of our knowledge, there is no evidence in the literature on the utility of regular surveillance of PICC to identify migration in newborn infants. Our study advances knowledge by showing that over their entire in situ duration PICCs migrate from their initial position at insertion. Therefore, performing radiographs may identify PICC migration. Perhaps this may also assist in identifying complications from PICC migration.

Interestingly, in our study, only 3.6% of PICCs migrated inwards and 15/643 (2.3%) of them from an initial central position, compared to 31% of upper limb PICCs reported by Srinivasan et al. [10]. Like Srinivasan’s study, most of the inward migrating PICCs were placed in the arm (78.3%). However, timing of radiographs and initial position of the PICC tips in Srinivasan's study and ours were different. Also, the arm position when taking radiographs may have contributed to this difference. We noted that the year of birth (insertion) impacted the position of the PICC tip independent of in situ duration. This may reflect improvement in PICC management practices over the study time from departmental quality improvement activities. These activities were consistency in educating PICC insertion procedure to the junior medical staff, consistency in the main proceduralist (nurse practitioner) for PICC insertion and better nursing PICC maintenance practice.

The strength of our study is inclusion of a large sample size. While our study bridges a gap in the literature, we acknowledge certain limitations. This was a retrospective single-centre observational study. However, the biases associated with this were reduced by using a prospectively maintained database for identification of patients and their medical records. There was an imbalance between the number of infants in the radiopaque agent used compared to radiopaque agent not used groups. As the decision on the use a radiopaque agent for PICC tip location was not based on random allocation but at the discretion of different providers, this may have introduced a selection bias. Consistent upper limb positioning was not practiced at the time of obtaining the radiographs. Although this may have contributed to PICC migration, only a fifth of PICCs were inserted in the arm. Future quality improvement activities could address inconsistencies in limb positioning while taking the radiographs. Complementary technology such as ultrasound was not used for PICC tip location. Finally, the results of our study should be interpreted with caution as it may not be applicable to all NICUs due to differences in PICC-related practices.

## Conclusion

Routine use of a radiopaque agent to identify PICC tip position may not be necessary. Perhaps its use could be reserved for when the PICC tip is not well visualized on a radiograph or when the PICC tip is in a non-central position. PICCs predominantly migrate outwards over their entire in situ duration. This can be monitored by weekly radiograph surveillance. This study has implications for reducing exposure to a radiopaque agent and migration surveillance practices. Future prospective quality improvement activities are needed to further refine these practices.

## Data Availability

All data generated or analysed during this study are included in this published article.

## Declarations

### Study approval statement

This research study was conducted retrospectively from data obtained for clinical purposes. Ethical approval was granted prior to study commencement by The Western Sydney Local Health District’s Human Research Ethics Committee, approval number 2112-01 QA.
